# Functional MRI Analysis of Brain Activity in Rats With Diabetic Bladder Dysfunction

**DOI:** 10.1111/cns.70466

**Published:** 2025-06-03

**Authors:** Mingzhuo Li, Xun Chen, JingJing Ye, Yudong Sun, Changhao Hou, Nailong Cao, Garth J. Thompson, Baojun Gu

**Affiliations:** ^1^ Department of Urology Shanghai Sixth People's Hospital Affiliated to Shanghai Jiao Tong University School of Medicine Shanghai China; ^2^ iHuman Institute ShanghaiTech University Shanghai China

**Keywords:** 5‐HT, ALFF, diabetic bladder dysfunction, fMRI

## Abstract

**Aims:**

This study aimed to investigate brain activity using functional magnetic resonance imaging (fMRI) in rats with 12‐week diabetic bladder dysfunction (DBD). Furthermore, as prior research has suggested that NLX‐112, a 5‐HT_1A_ receptor agonist, may alleviate DBD, we sought to explore its effects on brain activity in DBD rats.

**Methods:**

Male Sprague–Dawley rats were anesthetized with urethane and underwent cystometry alongside 9.4‐Tesla fMRI evaluations. Resting‐state fMRI was performed on empty bladders to compare baseline brain activity between groups. Gradient echo‐planar imaging was utilized to assess brain activation during micturition relative to relaxation, and a group analysis was conducted.

**Results:**

fMRI data from 12 diabetes mellitus (DM) rats and 12 normal control (NC) rats were analyzed. DM rats exhibited a significant reduction in the amplitude of low‐frequency fluctuation (ALFF) in the basal forebrain and cerebral cortex, a measurement of activity in brain networks. During micturition, DM rats showed increased activation in the thalamus, primary motor cortex, periaqueductal gray (PAG) and other regions. NLX‐112 administration did not significantly alter brain activity in DM rats.

**Conclusion:**

DBD rats exhibit heightened thalamic and PAG activity during micturition, potentially due to enlarged bladder capacity, with cortical activity serving as a compensatory mechanism. These findings highlight potential neural targets for DBD treatment.

## Graphical abstract

Using cystometry combined with 9.4‐Tesla fMRI evaluations, this study uncovered significant alterations in brain activity in diabetic bladder dysfunction (DBD) rats. Specifically, a reduction in ALFF was observed in the basal forebrain and cortex, while heightened activation was detected in the thalamus, periaqueductal gray, and primary motor cortex during micturition.

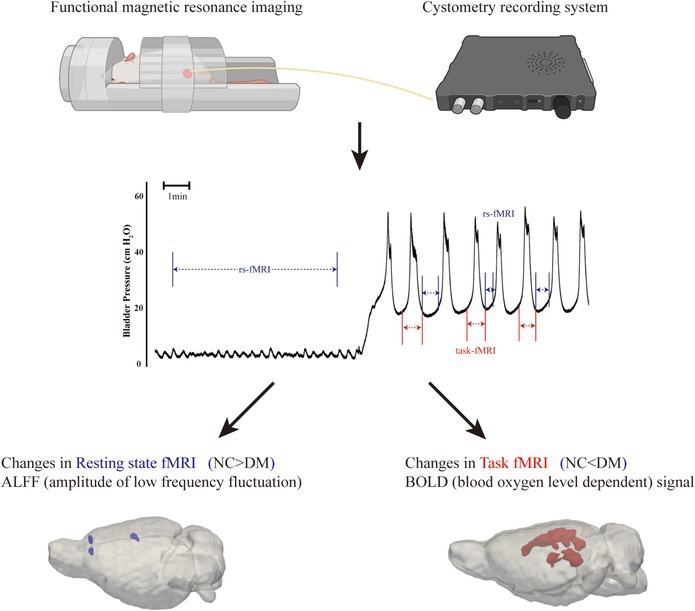

## 1 Introduction

Diabetes mellitus (DM) has been one of the most serious and common chronic diseases in our times. As of 2021, it was estimated that the global diabetes prevalence in 20–79‐year‐olds was estimated to be 10.5% (536.6 million people) [1]. DM is characterized by persistent hyperglycemia, leading to complications in multiple organs and posing significant challenges to healthcare systems [2]. Diabetic bladder dysfunction (DBD) is a common urological complication affecting over 50% of individuals with long‐standing, poorly controlled diabetes [3]. It is characterized by impaired bladder sensation, increased capacity, reduced contractility, and elevated postvoid residual volume [4].

Studying DBD in humans is challenging due to the heterogeneity of the diabetic population [5]. Traditionally, DBD is considered a two‐step process [6]. Initially, bladder hypertrophy and increased contractility occur as compensatory responses to polyuria. The second phase involves decompensation, attributed to nerve and muscle damage [7] caused by toxic metabolite buildup, oxidative stress, and other mechanisms [8]. Streptozotocin (STZ) is commonly used for the induction of DM in rats [9], with the decompensated phase typically emerging 9 to 12 weeks post‐STZ injection [6]. Urination involves the coordinated activity of smooth and striated muscles in the lower urinary tract (LUT), regulated by a complex neural network spanning the brain, spinal cord, and peripheral ganglia. Given the intricacy of these mechanisms, micturition is highly susceptible to injuries, diseases, and neurotoxic chemicals [10, 11]. The treatment of DBD poses a significant challenge in the field of urology. In our previous research, we found that 5‐HT_1A_ receptor agonists may act on the receptors in the dorsolateral nucleus of the L6‐S1 spinal cord, promoting periodic external urethral sphincter (EUS) activity and enhancing urethral relaxation, thereby improving the micturition efficiency of DBD rats [12, 13]. However, it remains unclear whether these related medications exert their effects on the brain.

Functional magnetic resonance imaging (fMRI) is a noninvasive technique using the blood oxygenation level‐dependent (BOLD) effect to study central nervous system function [14]. It can map task‐evoked brain activity or investigate spontaneous low‐frequency signal fluctuations during rest (resting‐state fMRI, rs‐fMRI). Rs‐fMRI employs indices like the amplitude of low‐frequency fluctuation (ALFF) and functional connectivity (FC): ALFF quantifies spontaneous neural activity changes associated with regional neural activity levels, while FC assesses interregional signal correlations. These metrics help characterize brain physiology and pathological alterations, such as in Alzheimer's disease or depression [15]. The task functional MRI (task‐fMRI) has been used to evaluate brain regions activation during micturition in other LUT diseases [16, 17, 18, 19]. However, past studies on DBD have primarily focused on the peripheral nervous system [5, 20, 21], with little known about central nervous system changes.

In this study, we employed a 9.4‐T fMRI to investigate alterations in the ALFF and FC within a rat model of 12‐week diabetes. We then correlated brain activation patterns with cystometric techniques to examine changes in the cerebral signal in diabetic rats. Furthermore, we delved deeper into exploring the cerebral modifications induced by the administration of 5‐HT_1A_ receptor agonists.

## 2 Materials and Methods

### 2.1 Animal Preparation

Forty male Sprague–Dawley rats, aged 8 weeks and weighing 280–320 g, were used in this study. They were randomly assigned to two groups: age‐matched normal controls (NC) and streptozotocin‐induced diabetes mellitus (DM), with 20 rats in each group. DM was induced following an 18‐h fast by a single intraperitoneal injection of 65 mg/kg STZ in 0.1 M citrate buffer (pH 4.5). NC rats received an equivalent volume of citrate buffer. Rats with blood glucose levels above 300 mg/dL, measured 72 h after STZ injection, were considered diabetic. All rats were housed on a 12‐h light/dark cycle with ad libitum food access. Three DM rats were excluded due to infection. Experiments were performed 12 weeks postinjection. Fifteen rats from the NC group and 14 from the DM group were used for intravesical recordings and MRI measurements, while three rats from each group were allocated for histological analysis. The entire procedure was approved by the animal care and use committee of Shanghai Sixth People's Hospital, in accordance with the policies of the Institutional Animal Care.

### 2.2 Drugs

Streptozotocin (STZ; Sigma‐Aldrich) was dissolved in 0.1 M citrate buffer (pH 4.5). The highly selective 5‐HT_1A_ receptor agonist NLX‐112 hydrochloride (Sigma‐Aldrich; pKi = 9) was dissolved in dimethyl sulfoxide (DMSO) and further diluted with distilled water.

### 2.3 Cystometrography Methods

Cystometrography was performed as previously described [22]. Rats were anesthetized with urethane (1.2 g/kg s.c.), under which neural activity patterns are relatively similar to those in awake conditions [23]. This dose provided long‐lasting anesthesia without the need for supplementation, a protocol validated in prior studies to maintain stable physiological parameters and intact bladder reflexes [16, 24, 25]. A polyethylene catheter (PE‐50; Becton Dickinson) was inserted into the left jugular vein for drug infusion. Through a midline abdominal incision, the bladder was accessed, and a polyethylene catheter (PE‐90; Becton Dickinson) with a flared end was inserted into the bladder dome and secured with a suture. The external end of the catheter was connected to a microinfusion pump (LD‐P2020 II; Lande Medical Instrument) and a pressure transducer (AD Instruments) via a three‐way valve for intravesical pressure (IVP) monitoring. Functional MRI scanning commenced 90–120 min after anesthesia administration [16], following confirmation of stable respiratory rate (65–75 breaths/min) and core body temperature (37.0°C ± 0.3°C via rectal probe).

### 2.4 Cystometric Protocol Combined With fMRI

Room temperature physiological saline was infused into the bladder to induce repeat voiding responses. The infusion rate was set at 0.2 mL/min for control rats and 0.8 mL/min for DM rats, the latter due to their enlarged bladders, ensuring enough voiding contractions within 8 min and 20 s of each MRI measurement. Bladder capacity was calculated by multiplying the first intercontraction interval by the infusion rate. Rats were placed inside a 9.4‐Tesla animal MRI scanner (Bruker BioSpec 94/30 USR) with a controlled temperature pad (37.8°C) and respiratory rate monitor (SA instruments). A 3*1 receiver array (Bruker) was used to enhance detection sensitivity. After a localizer scan for animal positioning, blood oxygenation level‐dependent (BOLD) fMRI was conducted using a gradient echo‐planar imaging (EPI) sequence (T2*‐weighted), with the following parameters: repetition time (TR) = 2000 ms; echo time (TE) = 16.195 ms; flip angle = 90°; matrix size = 128 × 96; field of view (FOV) = 3.5 × 3.5 cm^2^ (35 × 35 mm^2^); slice number = 25; slice thickness = 0.7 mm; in‐plane voxel size = FOV/matrix size = 0.27 × 0.36 mm^2^ (35 mm/128 × 35 mm/96); total raw voxel size = 0.27 × 0.36 × 0.7 mm^3^; total scan time: 8 min 20 s. T2‐weighted structural images were also obtained using a rapid acquisition with relaxation enhancement (RARE) sequence with the following parameters: TR = 2500 ms; TE = 34 ms; flip angle = 180°; matrix size = 256 × 256; field of view = 3.5 × 3.5 cm^2^; slice number = 25; slice thickness = 0.7 mm; total scan time: 2 min.

**FIGURE 1:**
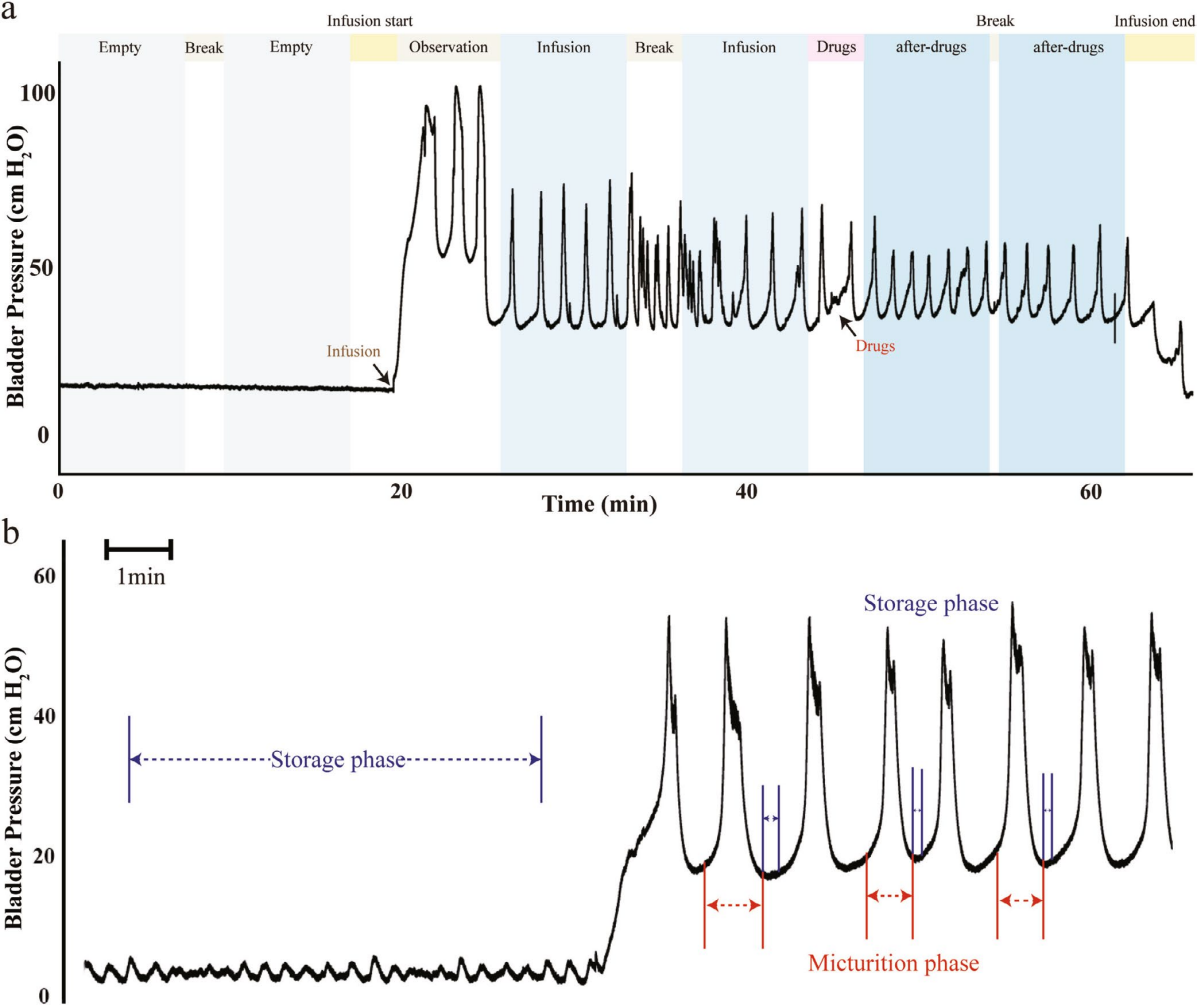
Experimental approach for fMRI during Reflexive Micturition. (a) This task‐related fMRI sequence began with an empty bladder, and MRI scanning was performed for two cycles as “Empty”. Then, the bladder infusion started. When stable bladder contractions were observed, MRI scanning was conducted for two cycles as “Infusion.” Next, MRI paused, and NLX‐112 (0.3 g/kg) was injected through the jugular vein. MRI scanning was performed for two cycles as “after – drugs.” Finally, MRI and infusion stopped. (b) The “Infusion” period was divided into the contraction period and the relaxation period by cystometric methods, and MRI images were divided into task‐fMRI and rs‐fMRI.

Firstly, an anatomical image (T2RARE) was acquired to facilitate anatomical localization and the registration of functional images. Then, as shown in Figure 1a, MRI scanning was performed twice with an empty bladder and twice with bladder infusion for each rat. NLX‐112 (0.3 g/kg) was injected, and postdrug injection MRI scanning was conducted twice in five rats from each group.

**FIGURE 2:**
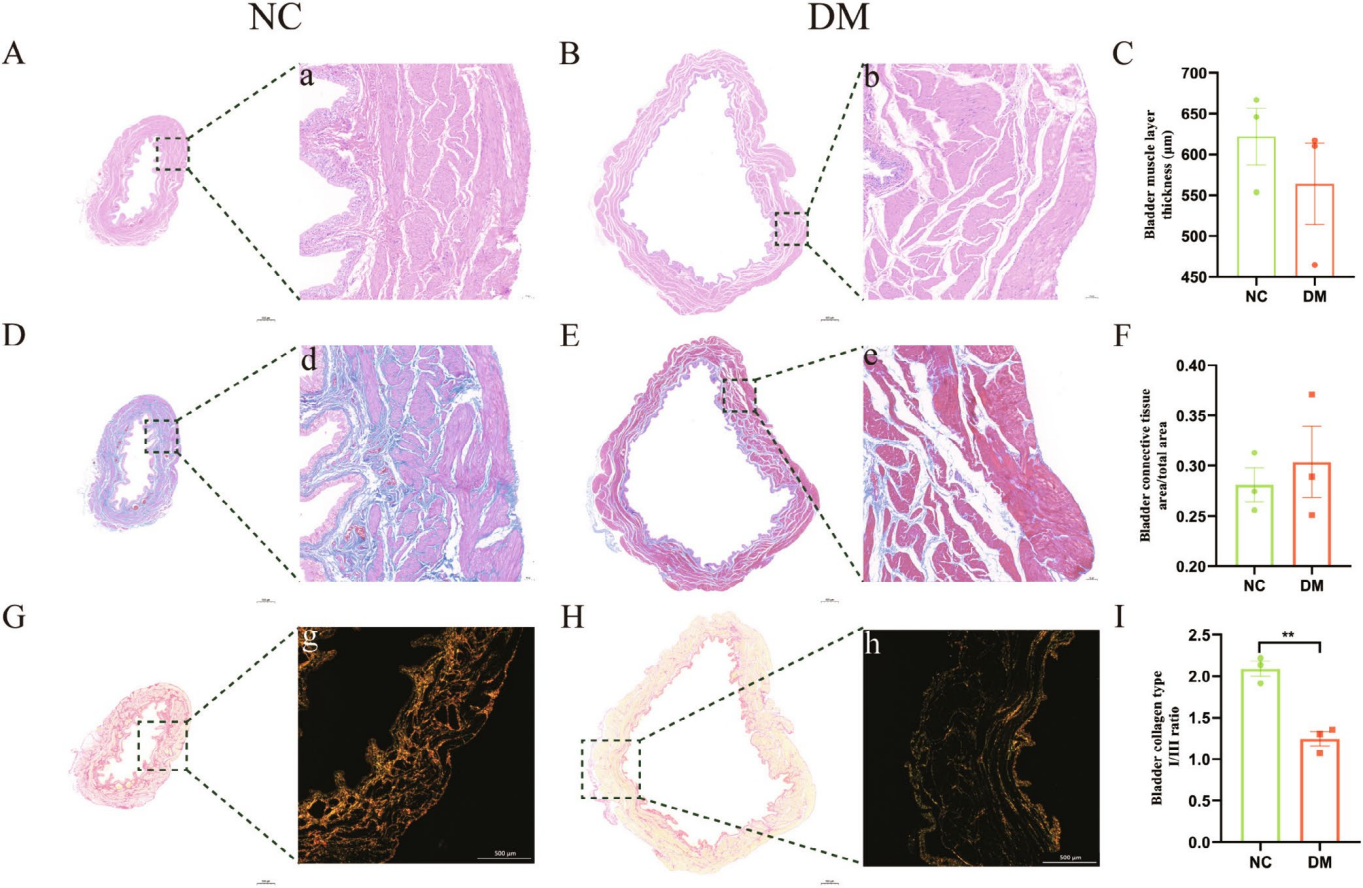
Histological analysis of bladder tissue in normal control (NC) rats (A, D, G) and 12‐week diabetic mellitus (DM) rats (B, E, H). Magnified images of the boxed areas in (A) are shown in (a), and so on (20×). (A, B) Hematoxylin and eosin‐stained images of bladder tissue. (D, E) Masson's trichrome‐stained images of bladder tissue. (G, H) Sirius red‐stained images of bladder tissue (type I collagen fibers: Red; type III collagen fibers: Pale yellow). Histograms (C, F, I) illustrate the differences in bladder muscle layer thickness (μm), bladder connective tissue area/total area, and bladder collagen type III/I ratio between the two groups.

Preprocessing steps included scaling the voxel size by a factor of 10, removing the first 10 volumes to allow for rat adaptation to the scanner, performing slice‐timing correction, realigning for head motion correction, normalizing to the standard rat brain atlas (SIGMA template) [26], and smoothing with an isotropic Gaussian kernel (full width at half maximum (FWHM) 8 mm) [27]. Data were excluded if head movements exceeded 1.0 mm of translation along the *x*, *y*, or *z* axes or 2.0° of rotation around any axis. To further reduce the effects of motion, the six motion signals from translation and rotation were then regressed from each voxel's time course.

### 2.5 Resting‐State Functional MRI Analysis

**FIGURE 3:**
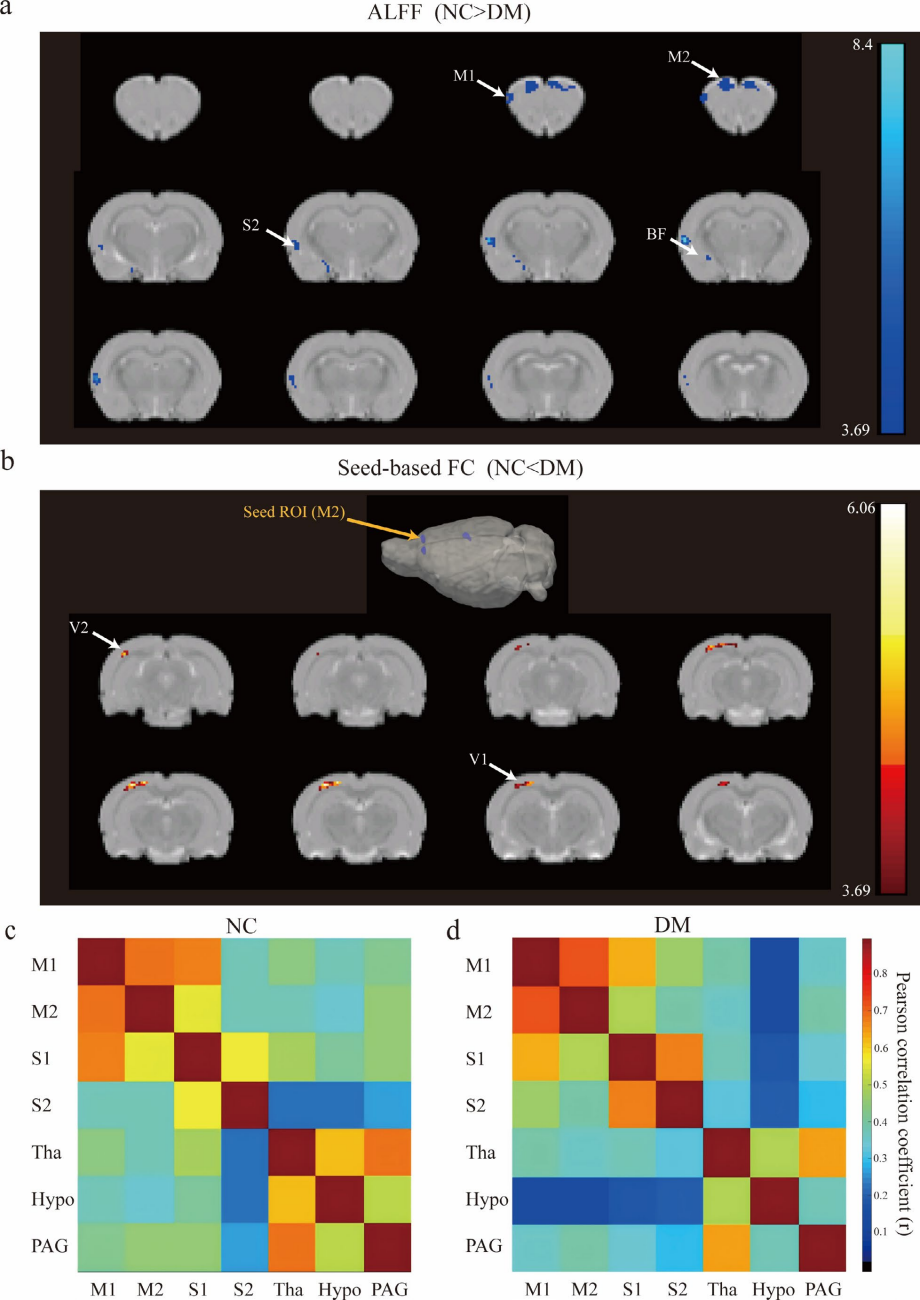
Comparison of the amplitude of low‐frequency fluctuations (ALFF) between diabetic (DM) and normal control (NC) rats, *N* = 12 for DM and NC rats. (a) Group comparison of ALFF. Significant brain regions (NC > DM, FDR corrected, T > 3.6862, *p* < 0.001) have been colored based on that region's *T* value and superimposed over the brain slice. Coronal slices are shown from rostral to caudal, with the top of the brain upward. (b) M2 was used as a seed, and brain regions with higher FC (NC < DM, FDR corrected, T > 3.6862, *p* < 0.001) have been colored based on that region's *T* value. (c,d) Mean Pearson correlation coefficient (*r*‐score) matrix of ROI in diabetic (DM) and normal control (NC) rats with empty bladders. FC, functional connectivity; ROI, region of interest; M1, primary motor cortex; M2, secondary motor cortex; S1, primary somatosensory cortex; S2, secondary somatosensory cortex; V1, primary visual cortex; V2, secondary visual cortex; BF, basal forebrain; Tha, thalamus; Hypo, hypothalamus; PAG, periaqueductal gray.

A total of 15 NC and 14 DM rats underwent BOLD fMRI imaging during continuous cystometry; however, 3 NC and 2 DM rats were discarded due to motion contamination. Finally, 12 NC rats and 12 DM rats were included in the comparative analysis of rs‐fMRI. fMRI data were preprocessed using Statistical Parametric Mapping (SPM12, Wellcome Trust Centre for Neuroimaging) and Data Processing Assistant for Resting‐State fMRI (DPARSF) on MATLAB R2014b. A 0.01–0.1 Hz band‐pass filter was then applied. ALFF was calculated on a per‐voxel basis for each rat. To compare ALFF values between the two groups, a *t*‐test was performed for each voxel (*p* < 0.001, cluster size > 10). Results were corrected for multiple comparisons using the False Discovery Rate (FDR) method to control for false positives. Significant voxels and regions were identified based on the corrected *p* value and cluster size threshold. The results were visualized as statistical maps highlighting brain regions with significant ALFF differences between the two groups. Using the region reported with different ALFF between groups as a seed, its correlation with the whole brain was calculated. Using Pearson's correlation coefficient, functional connectivity (FC) mapping was calculated on a per‐rat basis, and the average results are shown as heat maps in the figures (Figure 3c,d). The unpaired *t*‐test was used to investigate the differences in FC between the NC and DM groups (Figure S2). A voxel‐level height threshold of FDR corrected *p* < 0.001 and a cluster‐extent threshold of 10 contiguous voxels were considered statistically significant.

Region of interest (ROI) based FC was calculated for each rat. Seven ROIs with differences in ALFF or related to the lower urinary tract were selected and divided according to the SIGMA atlas, including primary motor cortex (M1), secondary motor cortex (M2), primary somatosensory cortex (S1), secondary somatosensory cortex (S2), thalamus (Tha), hypothalamus (Hypo) and periaqueductal gray (PAG). Pearson's correlation coefficients were calculated between each two of these regions. Results were presented as a connectivity matrix. An unpaired *t*‐test was used to compare FC matrices between groups. We used FDR for multiple comparison correction. As no connectivity pair survived the correction, we instead presented results as uncorrected *p* value together with averaged FC matrices from two groups.

### 2.6 Task Functional MRI Analysis

As shown in Figure 1b, the bladder contraction period was defined as the task state, and the storage period as the resting state. Due to the height difference between the MRI and urodynamic device and the tubing resistance, the pressure sensor detected transient pressure changes when the infusion pump switched from nonperfusion to perfusion. Since the rat bladders were not ligated at the neck, the intravesical pressure during noncontraction periods was the natural filling–phase pressure. These data were included in the rs–fMRI analysis to increase the dataset size.

As previously mentioned, 12 rats from each group were included in the analysis. First‐level analysis for each rat was performed using data from each task‐fMRI run with SPM12 (*n* = 7 per group). A classical general linear model (GLM) was applied to estimate the effect of bladder contraction. Functional MRI data were analyzed using GLM in SPM12 to characterize brain activity associated with bladder pressure changes. Preprocessing steps were the same as rs‐fMRI. Synchronized bladder pressure signals were resampled to the TR interval (2000 ms), low‐pass filtered (0.01–0.1 Hz), and integrated into the GLM design matrix as a continuous regressor convolved with a canonical hemodynamic response function (HRF; double‐gamma function, peak at 6 s, undershoot at 12 s). Motion parameters and cerebrospinal fluid/white matter signals were included as nuisance covariates to mitigate artifacts. Voxel‐wise statistical testing was performed using one‐sample *t*‐tests, and multiple comparison correction (FDR *p* < 0.05) defined the activation clusters. Group differences were assessed through a mixed‐effect second‐level analysis (T > 3.93). Additionally, bladder contraction periods before and after drug administration were compared similarly (*n* = 5 per group).

### 2.7 Histological Analysis

The rats were euthanized with an overdose of pentobarbital (100 mg/kg; Sigma, USA). Three rats from each group (NC and DM) were transcardially perfused with 4% paraformaldehyde, and the entire bladder was removed. Tissues were fixed in 10% neutral‐buffered formalin and embedded in paraffin. Transverse 4‐μm sections were cut using a paraffin microtome (RM2016; Leica) and mounted on coated slides. Every 10th section from the bladder and urethra of each NC and DM rat was stained with hematoxylin and eosin (H&E), Masson's trichrome, and Sirius red. Bladders were examined using a conventional light epifluorescence microscope (Nikon Eclipse Ti‐SR; Nikon, Tokyo, Japan), and images were digitally captured (Nikon DS‐U3). H&E‐stained images were used to measure total thickness and muscular layer thickness. Masson's trichrome‐stained images were analyzed for connective tissue area, total area, and muscle area. Sirius red‐stained images, viewed under a polarized light microscope, provided measurements of total collagen area and collagen types I and III. Tissue areas were measured using ImageJ 1.46 software (NIH, Bethesda, MD, USA).

### 2.8 Statistical Analysis

Data normality was assessed using the Shapiro–Wilk test for small sample sizes. For datasets meeting normality assumptions (*p* > 0.05), group comparisons were performed using unpaired *t*‐tests (reported as mean ± SD). Non‐normally distributed data were analyzed via Mann–Whitney U‐tests, with results presented as median [interquartile range]. Statistical significance was defined as *p* < 0.05. Data analysis and graph plotting were performed using GraphPad Prism (version 8.0.2).

## 3 Results

### 3.1 General and Histological Characteristics of NC and DM Groups

As shown in Table 1, 12 weeks postinduction, the body weight of DM rats significantly decreased compared to NC rats. Additionally, serum glucose levels were markedly higher in the DM rats. The DM rats also exhibited significantly larger bladder mass and capacity. However, there were no significant differences in bladder muscle layer thickness or the ratio of connective tissue area within the muscular layer between the two groups. Notably, the DM group had a higher collagen type III/I ratio than the NC group (Figure 2).

**TABLE 1:** Characteristics of normal control rats and rats with diabetes mellitus.

Characteristic	NC rats	DM rats	*p*
*N*	18	17	
Body mass, g	572.3 (17.33)	254.6 (26.66)	< 0.0001
Serum glucose, mg/ml	124.2 (13.53)	523.7 (24.26)	< 0.0001
Bladder mass, mg	99.68 (12.26)	222.7 (21.12)	< 0.0001
Bladder/body mass, mg/g	0.174 (0.02)	0.889 (0.16)	< 0.0001
*N*	12	12	
Bladder capacity, ml	1.088 (0.23)	2.741 (0.93)	0.0082
*N*	3	3	
Bladder muscle layer thickness, μm	622.2 (60.24)	564.0 (86.19)	0.3921
Bladder connective tissue area/total area in muscular layer	0.2810 (0.03)	0.3038 (0.06)	0.5926
Bladder collagen type I/III ratio	2.091 (0.16)	1.244 (0.15)	0.0024

*Note:* Data are expressed as mean (standard deviation).

Abbreviations: DM, diabetes mellitus; NC, normal control.

### 3.2 Resting‐State Functional MRI (Rs‐fMRI) in Diabetic Rats

Rs‐fMRI was used to investigate the difference in brain function between two groups. The ALFF [28] and FC [29] are two distinct fMRI methodologies that provide insights into voxel‐level local neural activity and voxel‐level functional integration, respectively. By averaging the values of all voxels within a specified brain region, it is possible to quantify the functional connectivity characteristics of that particular region. Consequently, we computed ALFF and FC maps to assess region‐specific alterations in resting‐state brain activity in diabetic rats. The basal forebrain (BF), secondary somatosensory cortex (S2), primary motor cortex (M1), and secondary motor cortex (M2) of the NC rats had significantly larger ALFF than those of the DM group (FDR corrected, T > 3.6862, *p* < 0.001) (Figures 3a and S3a, Table 2).

**TABLE 2:** Summary of resting‐state functional MRI in DM and NC rats.

			Talairach coordinates, mm				
	Brain region	Hemisphere	x	y	z	Peak intensity	Voxels
ALFF analysis (NC > DM)	Secondary somatosensory cortex	L	−70	−23	15	8.2764	18
	Secondary motor cortex	L	−16	55	48	4.0094	37
	Secondary motor cortex	R	11	55	51	4.0867	28
	Primary motor cortex	L	−43	55	36	3.9930	21
	Basal forebrain	L	−28	−29	−21	4.9307	11
Seed‐based FC (NC < DM)	Primary visual cortex	L	−51	−63	48	5.4470	17
	Secondary visual cortex	L	−42	−45	57	5.9497	23

Abbreviations: ALFF, amplitude of low‐frequency fluctuations, DM, diabetes mellitus, FC: functional connectivity, NC, normal control.

Next, we used the significant ALFF regions (M2) as a seed, and the whole brain FC was reported (Figures 3b and S3b, Table 2). When M2 was used as a seed, the primary visual cortex and secondary visual cortex showed a higher correlation with M2 in the DM groups. From the fMRI data, the Pearson correlation coefficients between the time courses of the regions of interest (M1, M2, S1, S2, thalamus (Tha), hypothalamus (Hypo), periaqueductal gray (PAG)) were calculated. There was a noticeable relatively larger correlativity (*r* > 0.7) between PAG and Tha in both groups than between other regions (Figure 3c,d). To explore the effects of DM on FC, we compared the FC of rats in the DM and NC groups. The correlation between Hypo and the cortex tended to be higher in the NC rats than in the DM rats (*p* < 0.05, uncorrected) (Figure S1).

### 3.3 Brain Activation During Reflexive Micturition in Two Groups

**FIGURE 4:**
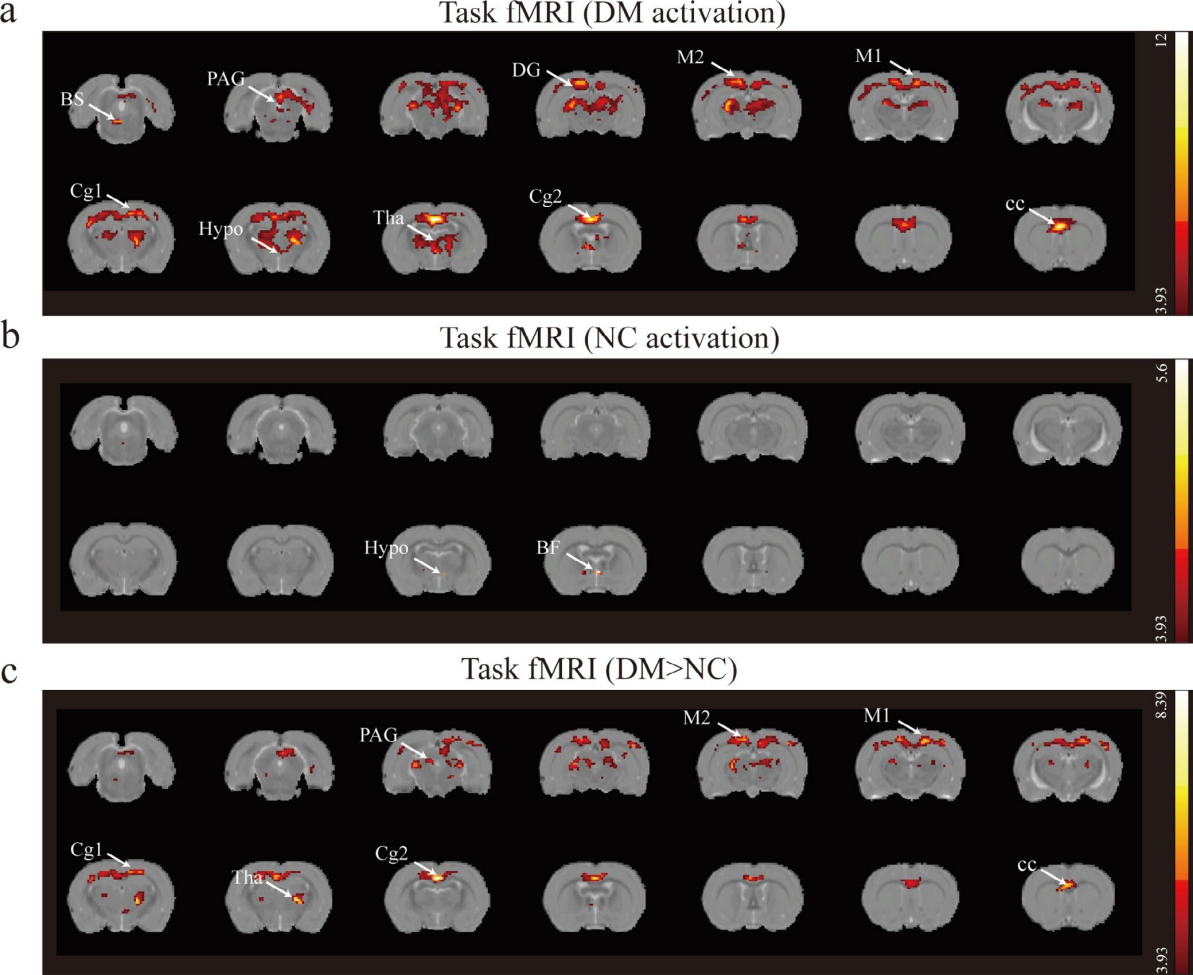
Urination induced BOLD positive activation in the brain, *N* = 7 for DM and NC rats. Significant brain regions have been colored based on that region's *T* value. Coronal slices are shown from caudal to rostral, with the top of the brain upward. (a) Functional group activation for diabetic rats, first‐level analysis. (b) Functional group activation for NC rats, first‐level analysis. (c) Second‐level analysis comparing a and b. Blobs in warm colors indicate regions of increased BOLD signal in diabetic rats compared with NC rats, including the thalamus, corpus callosum, primary cingular cortex, secondary cingular cortex, primary motor cortex, secondary motor cortex, and PAG (T > 3.93). BS, brainstem; cc, corpus callosum; DG, dentate gyrus; Cg1, primary cingular cortex; Cg2, secondary cingular cortex; M1, primary motor cortex; M2, secondary motor cortex; BF, basal forebrain; Tha, thalamus; Hypo, hypothalamus; PAG, periaqueductal gray.

Bulk movement often caused a global increase in raw image intensity across the brain, leading to whole‐brain activation during bladder contraction compared to the empty bladder. During urination, diabetic rats exhibited strong activations in the corpus callosum, thalamus, dentate gyrus, periaqueductal gray, secondary cingular cortex, primary cingular cortex, primary motor cortex, secondary motor cortex, and hypothalamus (Figure 4a and Table 3). The NC group showed activations in the basal forebrain and hypothalamus (Figure 4b and Table 3). When comparing brain activation between the two groups, diabetic rats showed stronger activations in the thalamus, dentate gyrus, superior colliculus, corpus callosum, secondary cingular cortex, primary motor cortex, secondary motor cortex, and PAG (FDR *p* < 0.05) (Figure 4c and Table 3). Moreover, after intravenous injection of NLX‐112 in diabetic rats, the primary somatosensory cortex showed stronger activations (*p* < 0.001, cluster size > 5 voxels, uncorrected) (Figure S2 and Table S1); however, multiple comparisons failed for the NLX‐112 test.

**TABLE 3:** Summary of brain activation areas during reflexive micturition.

	Brain region	Hemisphere	Voxels	Hemisphere	Voxels
DM	Brainstem	L	130	R	76
	Thalamus	L	447	R	427
	Corpus callosum	L	334	R	409
	Dentate gyrus	L	145	R	138
	Periaqueductal gray	L	128	R	100
	Secondary cingular cortex	L	104	R	100
	Primary cingular cortex	L	80	R	85
	Primary motor cortex	L	53	R	45
	Secondary motor cortex	L	22	R	24
	Hypothalamic	L	38	R	34
NC	Basal forebrain	L	6	R	5
	Hypothalamic	L	3	R	2
DM > NC	Thalamus	L	134	R	166
	Corpus callosum	L	201	R	283
	Secondary cingular cortex	L	71	R	67
	Primary cingular cortex	L	46	R	45
	Primary motor cortex	L	31	R	43
	Secondary motor cortex	L	16	R	18
	Periaqueductal gray	L	7	R	22

Abbreviations: DM, diabetes mellitus; NC, normal control.

## 4 Discussion

With the dramatic increase in diabetes incidence, there has been a corresponding rise in common urological complications, such as diabetic bladder dysfunction (DBD) [5, 21]. Streptozotocin (STZ)‐induced diabetic rats are extensively used as a rodent model for DBD [5]. In this study, bladder capacity in diabetic animals showed a significant increase at 12 weeks, remaining approximately 1.65 mL or 150% above control values. Collagens constitute the primary components of the extracellular matrix (ECM) in the LUT, with types I and III being the predominant subtypes influencing its biological function. Type I collagen imparts tensile strength to the tissue, whereas type III collagen is typically associated with striated fibrils in specific stromal matrices [30]. Consequently, the type I/III collagen ratio serves as an indicator of tissue tensile strength. Our findings demonstrated a significant reduction in the type I/III collagen ratio, potentially linked to DM‐induced downregulation of various ECM‐related genes, including those encoding type I and III collagen and fibronectin [31]. These findings are similar to those in previous reports in laboratory animals [6].

Increasing evidence indicates that the brain is a site of diabetic end‐organ damage [32]. DM is associated with cognitive impairment and functional decline. Although the pathogenesis of cerebral dysfunction in diabetes is not fully understood, research shows that hyperglycemia induces arterial stiffness, neuroinflammation [33], alterations in the blood brain barrier [34], and neurotransmitter imbalance [32, 35], resulting in compromised brain function. Previous studies have demonstrated decreased neural activity in specific brain regions, particularly the cerebral cortex, in diabetic animals compared to controls [33, 34, 36, 37, 38, 39, 40, 41]. Other brain regions, such as the striatal [36, 39, 41], caudate putamen [38], hippocampus [38], and insula [39], also showed functional impairments in different studies.

In this study, we observed significantly reduced amplitude of low‐frequency fluctuations (ALFF) in the cerebral cortex (M1, M2, S2) and basal forebrain of diabetic rats. The basal forebrain is rarely mentioned in diabetes‐related MRI studies, and discrepancies in findings may be due to small sample sizes and different stages of diabetes. Previous research suggests that the basal forebrain plays a significant role in cognitive and memory functions, and abnormalities in this region are linked to various neurodegenerative diseases [42, 43]. Epidemiologically, diabetes is associated with an increased prevalence of Alzheimer's disease (AD) [44] and Parkinson's disease (PD) [45]. The pathophysiological changes in the basal forebrain observed in this study suggest that ALFF, analyzed using rs‐fMRI techniques, could serve as an imaging biomarker for aberrant neural activity in diabetic animal models. This strategy may help further understand diabetes‐related neurodegenerative diseases, warranting additional research.

Recent static functional connectivity (FC) studies are dedicated to understanding the neural mechanism of cerebral dysfunction in DM individuals. In the prediabetic phase, increased FC was reported in both human [46, 47, 48] and animal studies [49], which may indicate a compensatory mechanism for reduced brain volume. In advanced diabetes, especially in patients with cognitive impairment, FC was significantly decreased in most studies. Damage was observed in specific connectivity between brain regions, including the bilateral lingual gyrus and sensorimotor cortex [50], caudate nucleus and precentral gyrus [51], and hippocampus and amygdala [52]. Moreover, in some literature, the thalamus contributed a lot to the diabetic cerebral FC dysfunction [53, 54], which was similar to our study. The results may help to better understand the changes in the central neural system due to DM. By Seed‐based FC, we observed increased FC between M2 and the visual cortex in DM groups—a novel finding without prior documentation in DM literature. While this association is unreported in diabetic neuropathology, studies in functional dystonia have demonstrated aberrant communication between the associative visual network and motor regions, suggesting that disrupted visuomotor integration may impair motor coordination [55]. We hypothesize that chronic hyperglycemia may induce subtle neural degeneration or adaptive rewiring within these cross‐modal pathways, potentially leading to preferential exaggeration of M2‐visual cortex connections as either a compensatory mechanism or pathological adaptation to sensorimotor dysfunction [56].

Previous studies have used isovolumetric bladder contractions to examine brain control in rats via fMRI [24, 25]. These contractions likely represent maximal bladder afferent input into the central nervous system but are less physiological due to urethral obstruction from ligation and bladder distension. In this study, we employed continuous cystometry to investigate brain control of the LUT. Unlike isovolumetric contractions, voiding induced by continuous infusion is more physiological as the urethra remains unobstructed [24]. However, the bladder filling time was insufficient to acquire high‐resolution data to detect neural activity [16]. Therefore, our analysis focused on brain activation during reflexive micturition. To enhance temporal resolution, we divided the bladder perfusion period into micturition and storage phases using urodynamic tests, which is an important method to evaluate lower urinary tract symptoms [57].

Current models of supraspinal LUT control, based on previous neuroimaging findings, suggest that ascending signals from the LUT are relayed through the periaqueductal gray (PAG), the pontine micturition center (PMC), and the thalamus to cortical areas involved in decision‐making and action‐taking processes [58, 59, 60]. The cortical areas controlling urination specifically comprise the insula, the prefrontal cortex (PFC), the anterior cingulate cortex (ACC) and other brain regions [58, 59, 60]. Our study demonstrated that bladder contraction in rats triggers brain activation changes similar to those observed in previous studies at large bladder volumes, specifically in the PAG and thalamus [58, 60]. We did not detect PMC activation during continuous cystometry, which aligns with previous findings that PMC activation has a smaller effect size and is barely detectable even with region of interest (ROI) analysis [16, 25].

In this study, DBD rats showed stronger activations in the thalamus, dentate gyrus, superior colliculus, corpus callosum, secondary cingulate cortex, M1, M2, and PAG compared to the NC group. Literature indicates that the primary motor cortex plays a crucial role in the descending control of urination via projections to the PMC [11], and agenesis of the corpus callosum is associated with hypocontractile neurogenic bladder [61]. We speculate that the larger bladder capacity in DBD rats leads to increased activity in the PAG and thalamus. However, the insula did not show strong signals to trigger the voiding reflex, resulting in compensatory stronger activity in the primary motor cortex and other brain areas. Preclinical evidence has demonstrated that 5‐HT_1A_ receptor agonists, such as NLX‐112, improve voiding efficiency in DBD models [12]. Notably, a pharmacological MRI (phMRI) study revealed that NLX‐112 elicits robust BOLD signal enhancements in the magnocellular preoptic nuclei (MCPO) and retrosplenial cortex, accompanied by pronounced hypoactivity in the orbital cortex and brainstem, with responses escalating in a dose‐dependent manner across tested concentrations [62]. In this study, the primary somatosensory cortex showed stronger activations, but multiple comparisons failed. This may be due to the small sample size (*n* = 5 per group) or the upregulation of 5‐HT_1A_ receptors in the dorsolateral nucleus in diabetic rats [13], suggesting that the drug may have more effects outside the brain.

This study has several limitations. First, the neural circuitry involved in bladder function is complex and widely distributed. While certain brain regions, such as the PMC, are closely linked to bladder functions, the smaller effect sizes in these areas prevented the detection of significant activation in the fMRI scans. Another limitation is the use of urethane anesthesia. While it is the best available anesthetic for maintaining the micturition response, it impairs bladder function [63]. On the other hand, anesthesia minimizes the influence of other brain processes, such as attention and emotion, which also likely affect voiding function.

## 5 Conclusion

This study utilized fMRI to examine the brain activity of rats with diabetic bladder dysfunction, incorporating continuous cystometry to explore the neurological changes associated with advanced stages of the condition. The results revealed that, in the urine storage phase, diabetic rats exhibited reduced activity in the cerebral cortex and basal forebrain. However, during the micturition period, these rats displayed heightened activity in several key brain regions, including the periaqueductal gray, thalamus, primary motor cortex, and corpus callosum. These findings offer valuable insights into the central nervous system's role in the pathophysiology of advanced diabetic bladder dysfunction, potentially paving the way for novel research directions aimed at understanding and treating this condition.

## Author Contributions

All authors contributed substantially to the study. **Mingzhuo Li**, **Xun Chen**, **Yudong Sun**, and **Changhao Hou** collected the data. **Mingzhuo Li** contributed to manuscript drafting. **JingJing Ye** and **Mingzhuo Li** performed data analysis. **JingJing Ye** and **Garth J. Thompson** designed the MRI methods. **Garth J. Thompson** and **Nailong Cao** revised the manuscript. **Baojun Gu** designed the experiments and revised the manuscript.

## Ethics Statement

The animal study was reviewed and approved by the Animal Care and Use Committee at Shanghai Jiao Tong University Affiliated Sixth People's Hospital (No: DWLL2024‐1048).

## Conflicts of Interest

The authors declare no conflicts of interest.

## Supporting information


Figures S1–S3.

**Table S1.** Summary of brain activation areas during reflexive micturition post‐NLX‐112 injection in diabetic rats.

## Data Availability

The data that support the findings of this study are available from the corresponding author upon reasonable request.
