# Supplementary material for: Functional MRI Analysis of Brain Activity in Rats With Diabetic Bladder Dysfunction
Source: CNS Neurosci Ther. 2025 Jun 3;31(6):e70466. doi: 10.1111/cns.70466 (PMC12130907; doi:10.1111/cns.70466)
Supplement: Supplementary file 1 — Figures S1–S3. [file CNS-31-e70466-s001.docx]

**Supplemental Material**


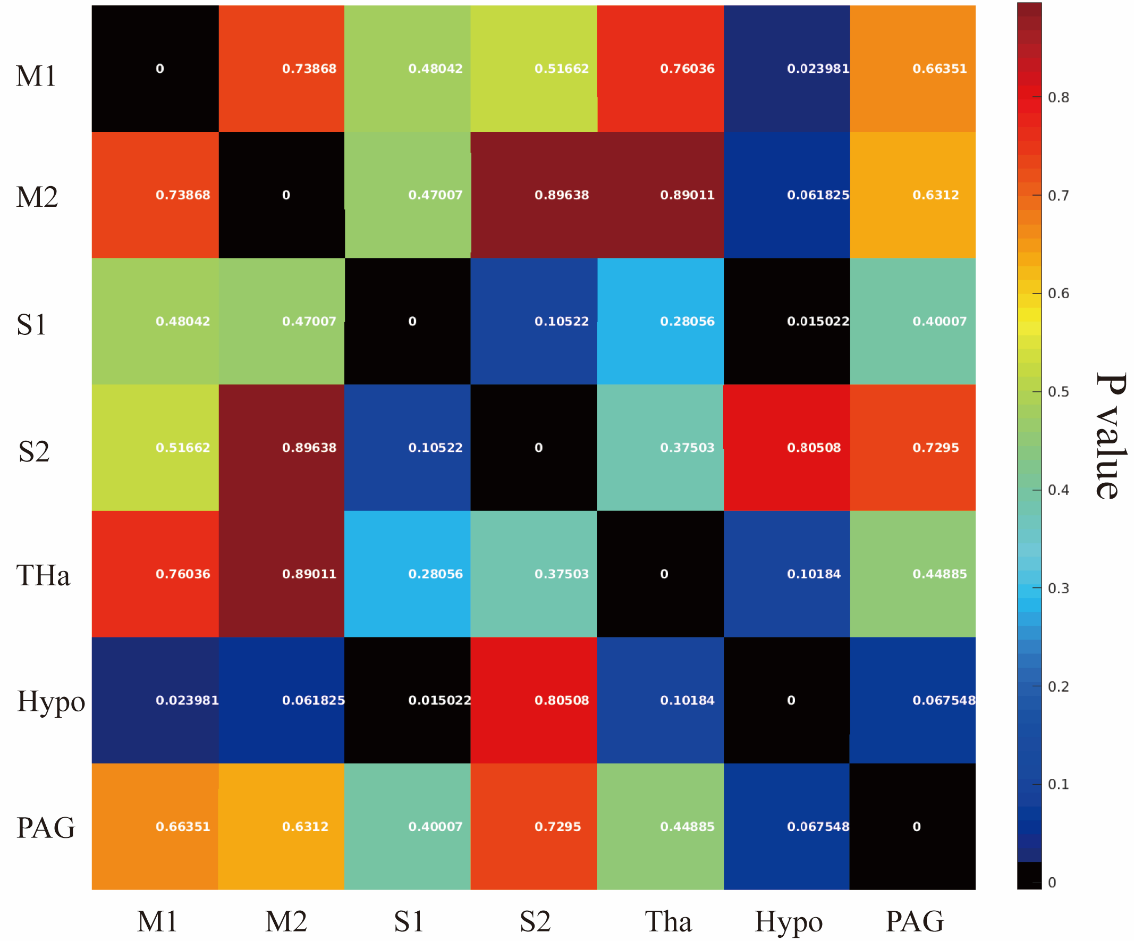


**Supplementary Figure S1** Comparison of the functional connectivity (FC) between Diabetic (DM) and Normal Control (NC) Rats, N = 12 for DM and NC rats (NC>DM, uncorrected). M1, primary motor cortex; M2, secondary motor cortex; S1, primary somatosensory cortex; S2, secondary somatosensory cortex; BF, basal forebrain; Tha, thalamus; Hypo, hypothalamus; PAG, periaqueductal gray.


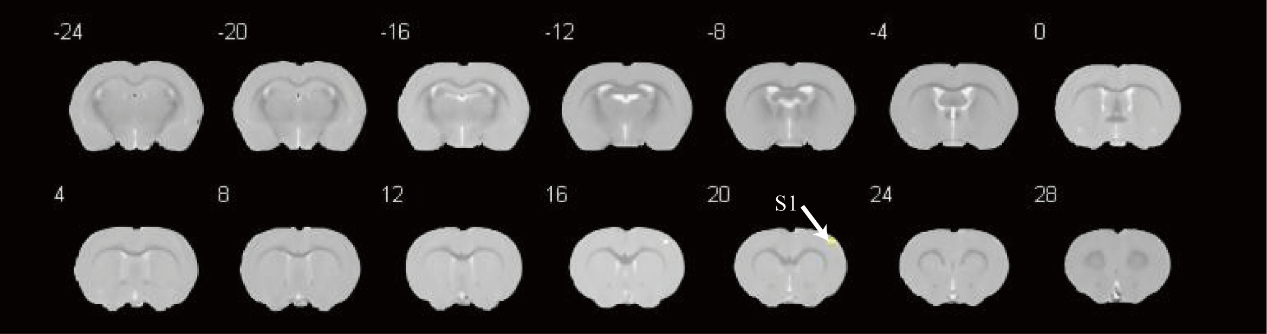


**Supplementary Figure S2** Urination induced BOLD positive activation in the brain post-NLX-112 injection in diabetic rats (n=5). Significant brain regions (T>3.93) have been colored based on that region’s T value. S1, primary somatosensory cortex.


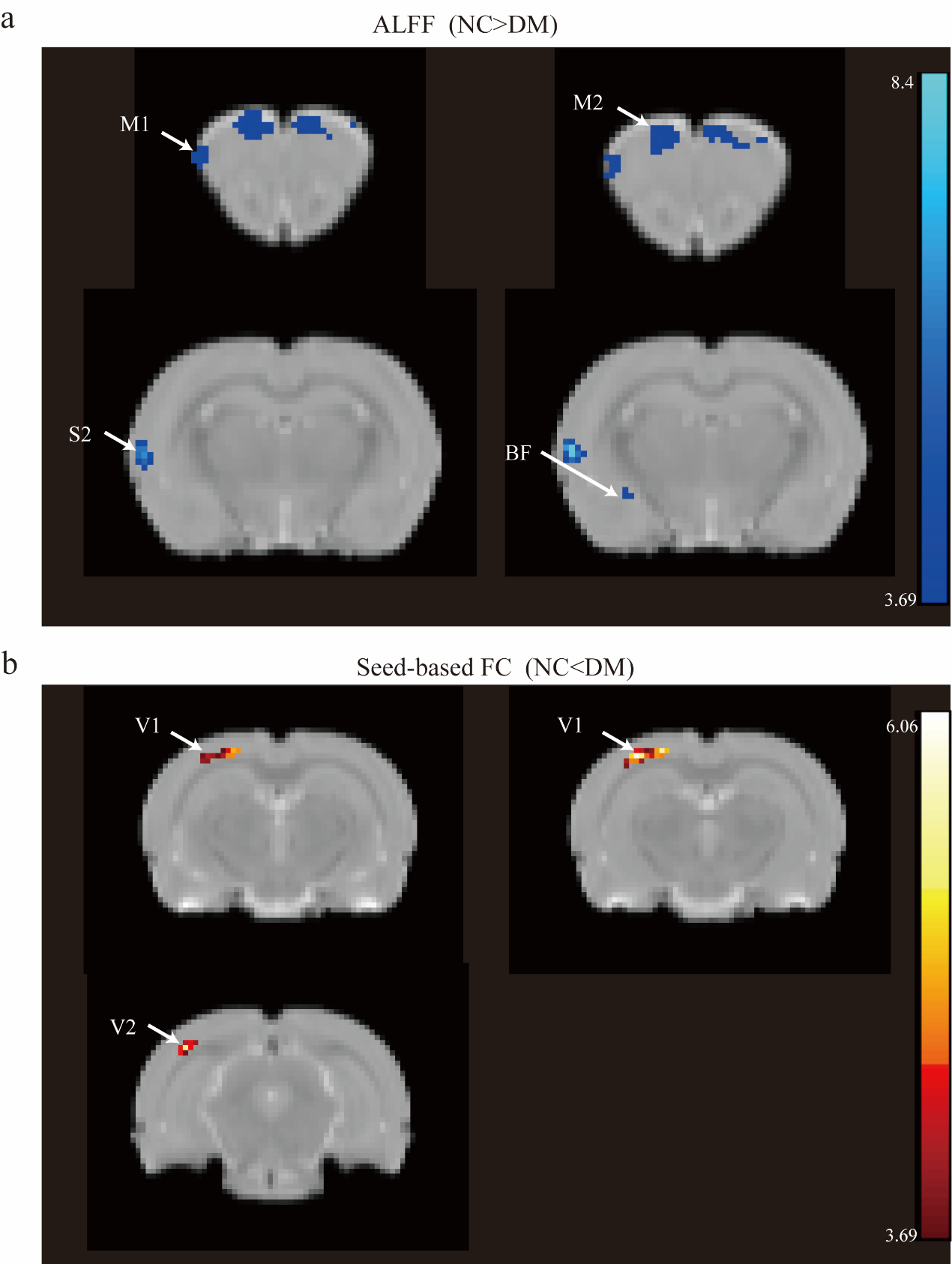


**Supplementary Figure S3** Magnified views of the slices in Fig. 3a and 3b. (a) is the magnified view of Fig. 3a, and (b) is the magnified view of Fig. 3b. FC, functional connectivity; M1, primary motor cortex; M2, secondary motor cortex; S2, secondary somatosensory cortex; BF, basal forebrain; V1, primary visual cortex; V2, secondary visual cortex.
