# Supplementary material for: Functional MRI Analysis of Brain Activity in Rats With Diabetic Bladder Dysfunction
Source: CNS Neurosci Ther. 2025 Jun 3;31(6):e70466. doi: 10.1111/cns.70466 (PMC12130907; doi:10.1111/cns.70466)
Supplement: Supplementary file 2 — Table S1. Summary of brain activation areas during reflexive micturition post‐NLX‐112 injection in diabetic rats. [file CNS-31-e70466-s002.docx]

Table S1. Summary of Brain Activation Areas During Reflexive Micturition post-NLX-112 Injection in Diabetic Rats.

|  |  | **Talairach coordinates, mm** | | |  |  |
| --- | --- | --- | --- | --- | --- | --- |
| **Brain region** | **Hemisphere** | **x** | **y** | **z** | **Peak intensity** | **voxels** |
| Primary Somatosensory Cortex | R | 49 | 19 | 48 | 9.5506 | 10 |
| Cornu Ammonis | L | -59 | -50 | 0.2 | 10.2161 | 5 |
| Primary Motor Cortex | R | 16 | -5 | 60 | 6.6814 | 4 |
